# Exploring the Genetic Diversity of the Jewel Beetles *Sternocera aequisignata* Saunders, 1866, and *S. ruficornis* Saunders, 1866 (Coleoptera: Buprestidae) in Thailand and Lao PDR

**DOI:** 10.3390/insects16030322

**Published:** 2025-03-19

**Authors:** Anisanee Thaenasa, Nakorn Pradit, Warayutt Pilap, Chavanut Jaroenchaiwattanachote, Komgrit Wongpakam, Khamla Inkhavilay, Jatupon Saijuntha, Wittaya Tawong, Warong Suksavate, Chairat Tantrawatpan, Weerachai Saijuntha

**Affiliations:** 1 Graduate School, Mahasarakham University, Maha Sarakham 44150, Thailand; thaenasa@gmail.com; 2 Walai Rukhavej Botanical Research Institute, Mahasarakham University, Maha Sarakham 44150, Thailand; nakorn.p@msu.ac.th (N.P.); warayutt@msu.ac.th (W.P.); komwongpa@gmail.com (K.W.); 3 Center of Excellence in Biodiversity Research, Mahasarakham University, Maha Sarakham 44150, Thailand; chavanut.j@msu.ac.th; 4 Center of Excellence in Biodiversity, National University of Laos, Vientiane 7322, Laos; khamla.inkhavilay@nuol.edu.la; 5 Faculty of Engineering, Mahasarakham University, Maha Sarakham 44150, Thailand; jatupons2534@gmail.com; 6 Department of Agricultural Sciences, Faculty of Agriculture Natural Resources and Environment, Naresuan University, Phitsanulok 65000, Thailand; wittayat@nu.ac.th; 7 Center of Excellence in Biodiversity, Center of Excellence in Research for Agricultural Biotechnology, Naresuan University, Phitsanulok 65000, Thailand; 8 Department of Forest Biology, Faculty of Forestry, Kasetsart University, Bangkok 10900, Thailand; wsuksavate@gmail.com; 9 Division of Cell Biology, Department of Preclinical Sciences, Faculty of Medicine, and Center of Excellence in Stem Cell Research and Innovation, Thammasat University, Rangsit Campus, Pathum Thani 12120, Thailand; 10 Biomedical Science Research Unit, Faculty of Medicine, Mahasarakham University, Maha Sarakham 44000, Thailand

**Keywords:** edible insect, genetic variation, cryptic species, genetic differentiation, conservation

## Abstract

Jewel beetles of the genus *Sternocera* are widely distributed across tropical regions, including Thailand and Lao PDR, where the green-legged *S. aequisignata* Saunders, 1866, and red-legged *S. ruficornis* Saunders, 1866, are commonly found. These jewel beetles have significant economic importance, as they are edible, and their iridescent wings are used to create jewelry, leading to high market demand and intensive harvesting. Additionally, their habitats are being rapidly destroyed, resulting in population decline. However, genetic information on these species remains limited. This study aims to investigate the genetic diversity of *S. aequisignata* and *S. ruficornis* from various localities in Thailand and Lao PDR using mitochondrial cytochrome c oxidase subunit 1 (*CO1*) and 16S ribosomal DNA (16S rDNA) sequences. High genetic diversity was observed, with 45 and 62 *CO1* haplotypes and 35 and 28 16S rDNA haplotypes identified in *S. aequisignata* and *S. ruficornis* populations, respectively. Haplotype network and phylogenetic analyses clearly distinguish *S. aequisignata* from *S. ruficornis*. Based on *CO1* sequences, *S. aequisignata* was divided into three distinct haplogroups (GG1–GG3). Haplogroup GG1 was the most widespread, occurring in both Thailand and Lao PDR, while haplogroups GG2 and GG3 were restricted to some localities in northern, western, and northeastern Thailand, as well as Lao PDR. These findings suggest the presence of cryptic diversity within *S. aequisignata*, with at least three genetically distinct groups. Further comprehensive studies on the biology, ecology, and genetic diversity of these jewel beetles across their distribution range are essential to better understand their evolutionary dynamics.

## Simple Summary

Jewel beetles play an important ecological and economic role, valued for their consumption and use in traditional crafts. However, habitat destruction and overharvesting threaten their populations, while genetic studies remain limited. Understanding their genetic diversity is essential for conservation. This study examines the genetic variation in two jewel beetle species, namely green-legged *Sternocera aequisignata* and red-legged *S. ruficornis* in Thailand and Lao PDR, using mitochondrial DNA markers. The results reveal high genetic diversity, with multiple haplotypes identified. Phylogenetic analysis can distinguish the species and uncover potential cryptic diversity, suggesting distinct genetic groups within one species. These findings highlight the complexity of this species’ genetic structure and the need for further research on their evolution and population dynamics. A better understanding of their genetics can support conservation efforts and sustainable management strategies.

## 1. Introduction

The jewel beetle is one of the largest and most diverse groups of the family Buprestidae, which comprises about 15,000 species in 522 genera worldwide [1]. In Thailand, there are over 300 different species of jewel beetles with color variation, ranging from bright metallic blue and green to black with small colored spots [2]. Currently, approximately 26 species of buprestid belonging to the genus *Sternocera* Eschscholtz, 1829, have been recognized worldwide [3]. Of these, only two species, i.e., green-legged *S. aequisignata* Saunders, 1866, and red-legged *S. ruficornis* Saunders, 1866, have been documented in Thailand, as well as throughout Southeast Asia [4]. The life cycle of *S. ruficornis* has been intensively studied by Pinkaew [4]. It starts with eggs deposited singly in the soil one centimeter deep at the base of their host plants. Each female laid around 5–12 eggs with an incubation period of approximately two months. The larva feeds on the rhizome of host plants, and then the last larva requires one year inside the earthen cell to transform into the pupal stage. Adults emerge from the soil after heavy rain during the rainy season and are active in the daytime. After mating, the female will oviposit the egg in the soil and die soon after [4].

The red-legged *S. ruficornis* commonly lives in large groups in dry dipterocarp forests in Thailand [4], while green-legged *S. aequisignata* does not [5]. There are two groups of host plants for *S. ruficornis*, one for the larval stage and the other for the adult stage. Larvae are found digging in the vicinity of *Arundinaria pusilla* and feeding on this root tree. Adult host plants are found in at least eleven trees, representing eleven species in nine genera from six families [2,4]. Their common predators include ants, which consume their eggs on the ground, as well as spiders and certain bird species [4]. Interestingly, *S. ruficornis* larvae do not damage the trees like other larvae in Burestidae, so they are not considered an economically important pest [4].

The jewel beetle *Sternocera* spp. is an economically important edible insect in Thailand. Their colorful wings in metallic dark green, copper green, bluish green, and golden green [2] also attract people to make jewelry and other marketable objects [4]. Due to the high market demand and the current inability to cultivate or farm them, their prices remain elevated. The commercials online and our survey in this study evidenced that the price of red-legged and green-legged jewel beetles in Thailand is approximately USD 0.15–0.3/individual, while adults containing eggs will be priced even higher, around USD 0.3–0.6/individual. In addition, its wings can be sold for USD 0.04–0.08/wing. After being crafted into jewelry, its market value ranges from USD 6 to 60 per piece. Subsequently, they are mass-hunted for sale, especially during the mating season. Also, their natural habitat and host plants are invaded and destroyed by human activities, such as urbanization, agriculture, deforestation, and forest fires, leading to a continuous decline in their population [6]. These reasons, including the lack of knowledge about their genetic diversity, population structure, and ecological requirements, mean that the conservation of these insects urgently needs to be addressed.

A thorough understanding of genetic diversity is essential for the sustainable utilization and conservation of jewel beetles. Research on the genetic diversity of the Buprestidae family has provided valuable insights into their evolutionary patterns and species differentiation. One notable example is *Agrilus viridis* (Linnaeus, 1758), which is a species complex within this family. Mitochondrial DNA analyses have revealed significant genetic divergence among lineages associated with different host plants, indicating cryptic diversity and suggesting that host specialization may drive speciation in this group [7]. To our knowledge, only a few studies of the biology, ecology, life cycle, and karyotype of the jewel beetle *S. ruficornis* in Thailand have been reported [4,8].

Previous studies successfully examined genetic variation and revealed the cryptic species of several edible insects in Thailand using mitochondrial DNA sequences [9,10]. The combination of mitochondrial cytochrome c oxidase subunit 1 (*CO1*) and large subunit ribosomal DNA (16S rDNA) allows for a more comprehensive genetic analysis by integrating both a rapidly evolving protein-coding gene and a more conserved ribosomal gene, enhancing the robustness of species identification, population differentiation, and phylogenetic reconstruction [9,10]. Thus, in this study, we aim to explore the genetic diversity of the jewel beetles, *S. ruficornis,* and *S. aequisignata*, collected from various localities in Thailand and Lao PDR using mitochondrial *CO1* and 16S rDNA as genetic markers.

## 2. Materials and Methods

### 2.1. Sample Collection and Molecular Analyses

**Figure 1:**
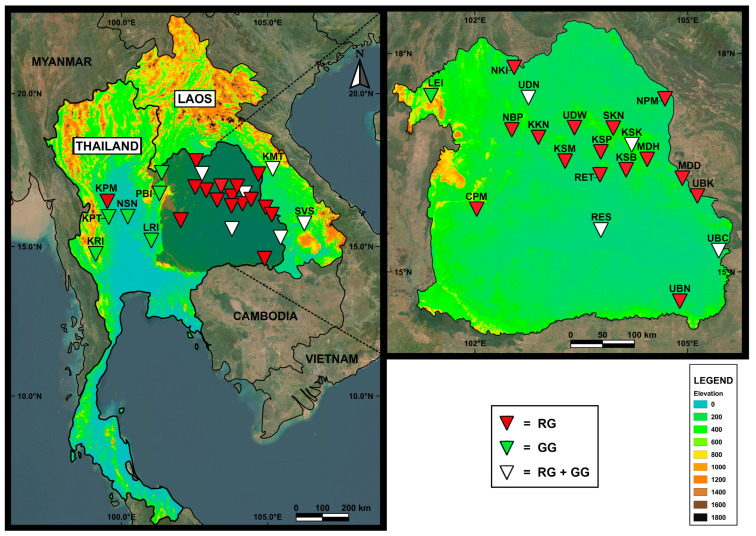
Map showing 28 sampling localities (see Table 1 for details) and the distribution of red-legged *Sternocera ruficornis* (RG) and green-legged *Sternocera aequisignata* (GG) in Thailand and Lao PDR. Localities marked with red triangles indicate collection sites where only *S. ruficornis* was found; green triangles indicate sites where only *S. aequisignata* was found; and white triangles indicate sites where both species were present.

The jewel beetles of green-legged *S. aequisignata* (GG) and red-legged *S. ruficornis* (RG) were collected from different localities in Thailand and Lao PDR by buying them from local markets or using a sweep net collected from their tree host. The sampling locality details are shown in Table 1 and Figure 1. Jewel beetle samples were kept in 80% ethanol until required for molecular analysis. These two species were differentiated based on leg coloration, specifically green and red, before undergoing DNA extraction. Total DNA was individually extracted from the left foreleg of each sample using the E.Z.N.A.^®^ Tissue DNA kit (Omega bio-tek, Norcross, GA, USA) following the manufacturer’s protocol. DNA samples were kept at −20 °C for further molecular analysis. Partial sequences of the *CO1* and 16S rDNA fragments were amplified and sequenced using primers and PCR conditions, as published by Pradit et al. [9]. The PCR products were electrophoresed in 1% agarose gels and visualized with the GelRed^TM^ Nucleic Acid Gel Stain (Biotium, Inc., Hayward, CA, USA). The amplified PCR product was cut and purified using the E.Z.N.A.^®^ Gel Extraction Kit (Omega bio-tek, Norcross, GA, USA). The purified PCR products were sent for DNA sequencing using the Sanger sequencing technique at ATGC Co., Ltd., Khlong Luang, Pathum Thani, Thailand.

### 2.2. DNA Sequence Analyses

All *CO1* and 16S rDNA sequences generated in this study were aligned using the ClustalW program version 1.4 [11] and edited by sight in the BioEdit program version 7.0.5.3 [12]. Molecular diversity indices and haplotype data were generated using the DnaSp v5 program [13]. The genetic difference between populations within a species was calculated based on *p*-distance [14] using the program MEGA XI [15]. The minimum-spanning haplotype networks were separately constructed using the *CO1* and 16S rDNA data of each species in the Network program version 10.2 based on a median-joining network [16] using all sequences generated in this study. Neutrality tests, including Tajima’s D and Fu’s Fs, Analysis of Molecular Variance (AMOVA), and Φ_ST_ analyses, were conducted using the Arlequin program version 3.5.2.2 [17].

### 2.3. Phylogenetic Tree Analyses

Phylogenetic trees were constructed using *CO1* and 16S rDNA sequences of *S. ruficornis* and *S. aequisignata* from Thailand and Lao PDR. Sequences of *Julodis variolaris* (Pallas, 1773) were used as the outgroup. Maximum likelihood (ML) phylogenetic analysis was performed using the General Time Reversible model with gamma distribution and invariant sites (GTR + G + I model) [18] while neighbor-joining (NJ) trees [19] were also constructed. Both analyses were conducted using MEGA XI [20], with nodal support estimated by 1000 bootstrapping replicates.

### 2.4. Delineation of Genetic Groups

Three single-locus species delimitation methods—Automatic Barcode Gap Discovery; ABGD [21], Assemble Species by Automatic Partitioning; ASAP [22], and Poisson Tree Processes; PTP [23]—were applied to both markers for the genetic lineage delineation of both species. For ABGD, analyses were conducted using the web server (https://bioinfo.mnhn.fr/abi/public/abgd/abgdweb.html; accessed on 16 January 2025). The Kimura (K80) substitution model was chosen, with the maximum (Pmax) and minimum (Pmin) intraspecific distances set to their default value of 0.001. The barcode gap width was set to 1.5. Recursive partitions were examined, with a prior maximal distance of P = 7.74 × 10^−3^. ASAP analysis was performed using the online tool (https://bioinfo.mnhn.fr/abi/public/asap/; accessed on 16 January 2025). The species partition with the lowest ASAP score and a suitable threshold distance (dT) was selected under the Kimura (K80) model. Default parameters were used, with a transition/transversion ratio (ts/tv) of 2.0 and a minimum and maximum threshold distance of 0.05 to 0.5. The PTP analysis was conducted using a web server (https://mptp.h-its.org/#/tree/; accessed on 18 January 2025). The ML trees for the *CO1* and 16S rDNA genes generated in MEGA XI [20] were used as input files. All parameters were kept at default, employing a single threshold (*p* = 0.001) for delimitation.

## 3. Results

### 3.1. Molecular Diversity Indices

#### 3.1.1. Green-Legged *S. aequisignata*

The 67 and 80 samples of green-legged *S. aequisignata* were successfully amplified for the *CO1* and 16S rDNA gene, which were deposited in GenBank under accession numbers PQ132856–PQ132900 and PQ132966–PQ133000, respectively. Based on multiple alignments of a 627 bp *CO1* sequence, 105 (16.7%) variable sites were observed (Table S1) and divided into 45 haplotypes (GgC1–GgC45). Of these, 39 haplotypes were uniquely found in a particular area, while the other 9 haplotypes were shared across different areas (Table 2). Genetic diversity indices analysis showed that the average haplotype and nucleotide diversity of *CO1* ranged between 0.983 ± 0.006 and 0.0361 ± 0.0020, respectively. Based on 475 bp 16S rDNA sequences analysis, 29 variable sites (6.1%) were observed (Table S2) and 35 haplotypes (GgR1–GgR35) were defined. Of these, 25 haplotypes were uniquely found at a particular locality/population, while the other 10 haplotypes were shared between populations. Genetic diversity indices analysis showed that the average haplotype and nucleotide diversity of 16S rDNA ranged between 0.958 ± 0.010 and 0.0116 ± 0.0004, respectively (Table 2).

#### 3.1.2. Red-Legged *S. ruficornis*

The 125 and 161 samples of red-legged *S. ruficornis* were successfully amplified for the *CO1* and 16S rDNA gene, which were deposited in GenBank under accession numbers PQ132794–PQ132855 and PQ132938–PQ132965, respectively. Based on 627 bp *CO1* sequence analysis, 107 (17.1%) variable sites were observed (Table S1) and divided into 62 haplotypes (RgC1–RgC62). Of these, 54 haplotypes were uniquely found in a particular area, while the other 9 haplotypes were shared across different areas (Table 3). Genetic diversity indices analysis showed that the average haplotype and nucleotide diversity of *CO1* ranged between 0.974 ± 0.012 and 0.0174 ± 0.0007, respectively. Based on 475 bp 16S rDNA sequences analysis, 29 variable sites (6.1%) were observed (Table S2), and 28 haplotypes (RgR1–RgR28) were defined. Of these, 25 haplotypes were uniquely found at a particular locality/population, while the other 3 haplotypes were shared between populations. Genetic diversity indices analysis showed that the average haplotype and nucleotide diversity of 16S rDNA ranged between 0.706 ± 0.029 and 0.0030 ± 0.0003, respectively (Table 3).

### 3.2. Neutrality Test

#### 3.2.1. Green-Legged *S. aequisignata*

Neutrality tests for *S. aequisignata* based on CO1 sequences showed Tajima’s D values ranging from −0.88793 to 0.86284 (mean = −0.04774), with no significant deviations from neutrality (all *p*-value > 0.05). Negative values suggest potential population expansion or purifying selection, while positive values indicate balancing selection or population structure. Fu’s FS values ranged from −1.76449 to 5.52935 (mean = 0.71043), with no significant results, implying no strong evidence for demographic changes. For 16S rDNA sequences, the mean Tajima’s D was −0.1358 (±0.5623), indicating mostly neutral evolution. Fu’s FS had a mean of −0.5943 (±1.4484), with a significantly negative value in the UBC population (−3.15612, *p*-value = 0.009), suggesting recent population expansion (Table S3).

#### 3.2.2. Red-Legged *S. ruficornis*

Neutrality tests based on mitochondrial *CO1* and 16S rDNA sequences of *S. ruficornis* populations were conducted to assess deviations from neutrality, indicating potential demographic history or selection. Tajima’s D values varied among populations, with significantly negative values in KSM (−1.4774, *p*-value = 0.019) and UDW (−1.7411, *p*-value = 0.014) for *CO1* and UBC (−1.7137, *p*-value = 0.03) for 16S rDNA, suggesting population expansion or purifying selection. Fu’s FS test further supported expansion, with significantly negative values in KSK (−2.7081, *p*-value = 0.021), KSP (−6.2566, *p*-value = 0.000), and UBN (−2.8138, *p*-value = 0.024) for *CO1*, and UBC (−2.1637, *p*-value = 0.026), UBN (−1.7836, *p*-value = 0.045), and UDN (−2.1569, *p*-value = 0.023) for 16S rDNA. In contrast, some populations exhibited positive but non-significant neutrality test values, indicating possible genetic drift or balancing selection (Table S3). Overall, the results suggest recent population expansion in several populations, while others have remained stable or experienced genetic drift.

### 3.3. Haplotype Network

**Figure 2:**
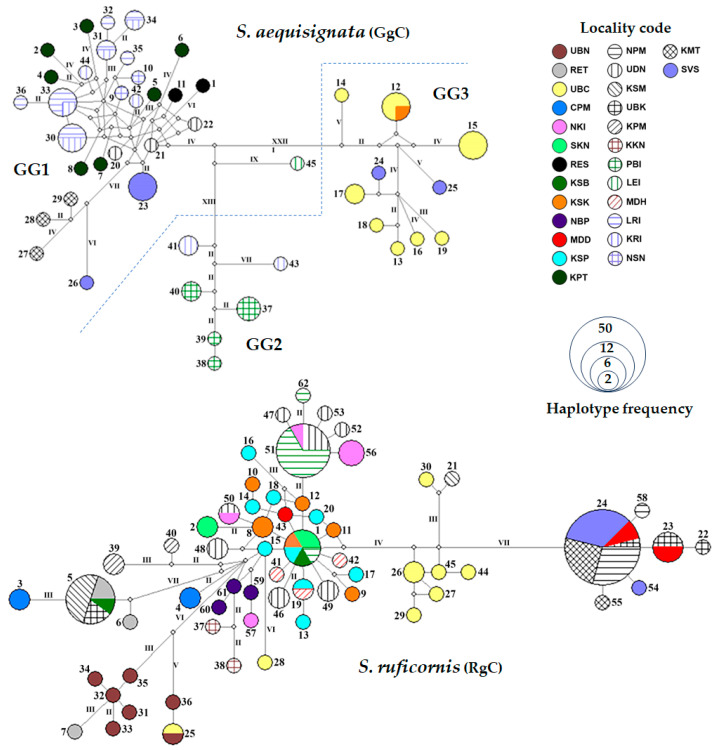
Minimum spanning haplotype network constructed from *CO1* haplotypes of *Sternocera aequisignata* (GgC) and *S. ruficornis* (RgC) from Thailand and Lao PDR. Three haplogroups of *S. aequisignata*, namely GG1, GG2, and GG3 were classified. Different printed patterns and colors in the haplotype networks represent the various localities examined in this study. The size of each circle reflects the proportion of specimens associated with each haplotype. The length of each branch is indicated by Roman numerals representing the number of mutational steps (ms), with values greater than one displayed. Locality codes are provided in Table 1.

All *CO1* and 16S rDNA sequences of *S. aequisignata* and *S. ruficornis* samples were analyzed separately to generate the haplotype networks. Based on 16S rDNA sequence analysis, no haplogroups were identified in either species, as the maximum difference observed was only two mutational steps (Figure S1). However, using a threshold of more than 10 mutational steps per branch as the criterion for haplogroup classification, the CO1 haplotype network of *S. ruficornis* did not form distinct haplogroups, as the maximum number of mutational steps observed was seven (Figure 2). In contrast, the CO1 haplotype network of *S. aequisignata* revealed three haplogroups. Haplogroup GG1 included 28 haplotypes (GgC1–GgC11, GgC20–GgC23, GgC26–GgC36, and GgC42) and was found across all localities except PBI, LEI, UBC, and KSK. Haplogroup GG2 consisted of six haplotypes (GgC37–GgC41, and GgC43) from the KRI and PBI populations. Haplogroup GG3 comprised ten haplotypes (GgC12–GgC19, GgC24, and GgC25) from the UBC, KSK, and SVS populations (Figure 2). Notably, one haplotype (GgC45) from LEI exhibited more than 10 mutational steps of difference from all the other haplotypes. However, since this was represented by a single sample, we have not yet classified it as a separate haplogroup (Figure 2).

### 3.4. Genetic Differences and Genetic Structure Analyses

All *CO1* and 16S rDNA sequences from *S. aequisignata* and *S. ruficornis* samples were used to calculate the genetic differences (*p*-distance) within each species (Tables S4 and S5). The interspecific genetic variation between populations of *S. aequisignata*, based on *p*-distance calculated from 16S rDNA and *CO1* sequence analyses, ranged from 0.0025 to 0.0206 and 0.0072 to 0.0627, respectively (Table S4). The UBC and KSK populations in haplogroup GG2 of *S. aequisignata* showed the greatest genetic differences from the other populations, with *CO1 p*-distances ranging from 0.0105 to 0.0627 (Table S4). Similarly, the PBI population of *S. aequisignata* in haplogroup GG3 exhibited a high genetic difference, with *p*-distances ranging from 0.0378 to 0.0520 (Table S4). By contrast, the interspecific genetic variation between populations of *S. ruficornis*, based on *p*-distance calculated from 16S rDNA and *CO1* sequence analyses, ranged from 0.0025 to 0.0206 and 0.0000 to 0.0115, respectively (Table S5). Meanwhile, the UBN population of *S. ruficornis* exhibited the greatest genetic differences from other populations, with *CO1 p*-distances ranging from 0.0205 to 0.0342 (Table S5). However, the 16S rDNA *p*-distance showed no significant differences when comparing all populations within each species.

**Figure 3:**
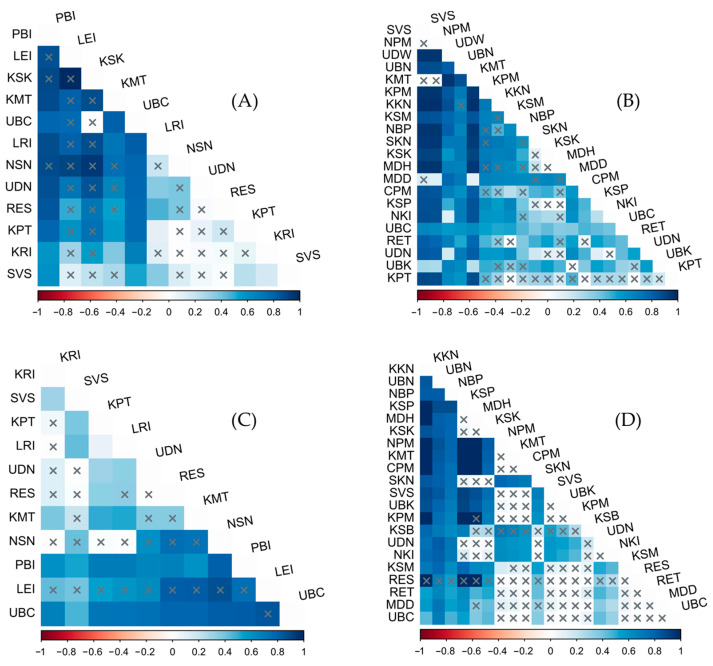
Heat map showing genetic differences represented by Φ_ST_ values based on *CO1* sequences among populations of *Sternocera aequisignata* (**A**) and *S. ruficornis* (**B**); genetic differences based on 16S rDNA sequences among populations of *S. aequisignata* (**C**) and *S. ruficornis* (**D**). The x-axis represents Φ_ST_ values ranging from −1 to 1, corresponding to variations in color shedding. The y-axis represents the locality codes of jewel beetle populations. Cross marks (x) indicate no significant difference (*p*-value ≥ 0.05), while other values represent significant genetic differences (*p*-value < 0.05). Locality codes are provided in Table 1.

Genetic differences were observed based on Φ_ST_ analysis; comparisons among populations of *S. aequisignata* revealed that the PBI and UBC populations were the most genetically distinct from other populations, with significant differences observed in both *CO1* and 16S rDNA sequences (Figure 3). For *S. ruficornis*, the SVS, NPM, UDW, UBN, and KMT populations exhibited significant genetic differences from most other populations based on *CO1* sequences. In contrast, 16S rDNA sequences indicated that the KKN, UBN, and NBP populations were significantly different from the majority of the other populations. Additionally, several other population pairs of both *S. aequisignata* and *S. ruficornis*, analyzed using *CO1* and 16S rDNA sequences, also exhibited significant genetic differences (Figure 3).

The analysis of genetic variation revealed significant differentiation at all hierarchical levels, with the majority of genetic variation occurring among the defined groups. AMOVA analysis of the genetic structure of *S. aequisignata* populations, based on *CO1* sequences, demonstrated significant genetic differentiation among the three defined genetic groups (GG1–GG3), with *F*_CT_ = 0.73059 (*p*-value < 0.001). This finding aligns with the genetic clustering inferred from haplotype network analysis. Additionally, significant genetic variation was detected among populations within groups (*F*_SC_ = 0.30545, *p*-value < 0.001) and among individuals within populations (*F*_IS_ = 0.81288, *p*-value < 0.001) (Table 4). These results indicate that genetic differentiation is most pronounced between groups, with moderate differentiation among populations within groups and substantial variation within populations.

### 3.5. Phylogenetic Trees

**Figure 4:**
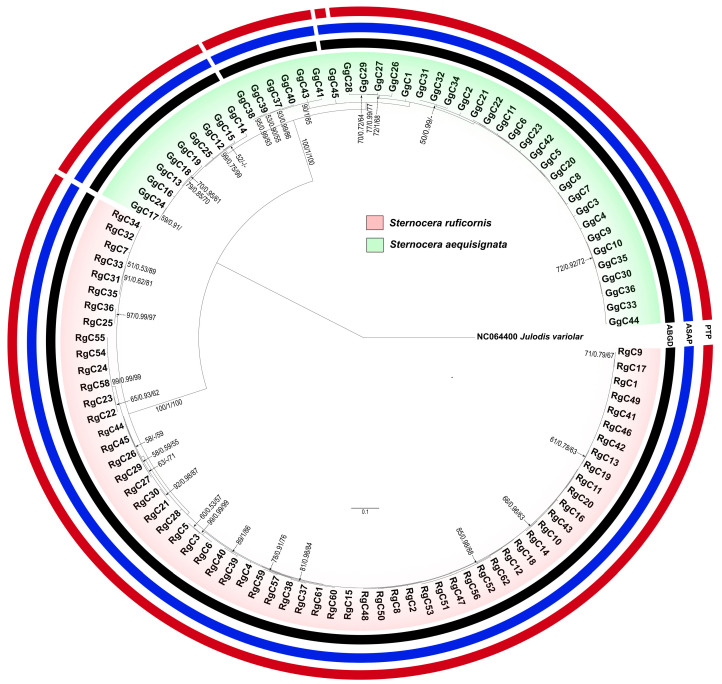
Phylogenetic tree constructed from *CO1* sequences of *Sternocera aequisignata* (GgC) and *S. ruficornis* (RgC). Bootstrap values for maximum likelihood (ML) and Bayesian inference (BI), as well as posterior probabilities for neighbor-joining (NJ), are indicated above or near the branches. The scale bar represents 0.1 substitutions per nucleotide position. Each colored circle represents a species delimitation method: the red circle indicates the Poisson Tree Processes (PTP); the blue circle represents Assemble Species by Automatic Partitioning (ASAP); and the black circle denotes Barcode Gap Discovery (ABGD) methods. Different genetic groups are indicated by gaps within each circle.

Based on 16S rDNA and *CO1* sequences, the phylogenetic trees clearly showed distinct lineages of *S. aequisignata* and *S. ruficornis* (Figure 4 and Figure S2). The PTP, ASAP, and ABGD analyses supported the presence of three cryptic genetic groups of *S. aequisignata* based on *CO1* sequences, together with high bootstrap values and strong Bayesian inference (BI) support (Figure 4). These groups correspond to the three haplogroups (GG1–GG3) identified in the haplotype network analysis (Figure 2). However, the 16S rDNA phylogenetic tree did not support this grouping (Figure S1). Additionally, one sample of *S. aequisignata* from Loei Province (haplotype GgC45) was separated from the others in the PTP analysis of the *CO1* tree (Figure 2), consistent with the haplotype network analysis. In contrast, the phylogenetic trees of *S. ruficornis* did not provide a reason behind the distinct genetic groups in either the 16S rDNA or *CO1* analyses (Figure 2 and Figure S2).

## 4. Discussion

We recently found evidence for the distribution of *S. ruficornis* mostly in northeastern Thailand and Lao PDR, while *S. aequisignata* is commonly found in the northern and central regions of Thailand. While the co-existence of *S. aequisignata* and *S. ruficornis* has been found to occur in some areas in northeast Thailand and Lao PDR, our findings support the previous report that *S. ruficornis* is commonly found in dry dipterocarp forests, while *S. aequisignata* is not [4]. However, *S. aequisignata* has been found in some areas in northeastern Thailand and Lao PDR. Vice versa, *S. ruficornis* was also found in a central region. This finding suggests that the habitat of these two species is not completely separated; thus, further exploration of the habitat range of these jewel beetles in Southeast Asia is necessary.

To our knowledge, there is very limited information on the genetic variation in the edible jewel beetles, particularly those in the genus *Sternocera* in Southeast Asia. Therefore, this study represents the first comprehensive investigation of the genetic variation found in two species in this genus, namely *S. ruficornis* and *S. aequisignata*, in Thailand and Lao PDR. We found remarkably high genetic diversity, with a high frequency of unique haplotypes in each population. This finding provides evidence that the natural populations of *S. aequisignata* and *S. ruficornis* in the studied areas exhibit high genetic diversity, suggesting a high rate of genetic recombination (gene flow). Similar findings have been reported in several edible insects in Thailand and Lao PDR, for example, the large brown cricket *Tarbinskiellus portentosus* (Lichtenstein 1796) [9], giant water bug *Lethocerus indicus* (Lepelletier and Serville, 1825) [10], and mole cricket *Gryllotalpa orientalis* Latreille, 1802 [24].

Interestingly, we observed a significant difference only in *S. aequisignata* populations, which were categorized into three *CO1* haplogroups: GG1, GG2, and GG3. Haplogroup GG1 was predominantly associated with populations from northern, central, and western Thailand, including two localities in Lao PDR. Haplogroup GG2 was specifically found in PBI (northern Thailand) and some samples from KRI (western Thailand). In contrast, haplogroup GG3 was restricted to northeastern Thailand, including SVS in Lao PDR. This genetic structuring likely reflects historical biogeographical barriers, such as rivers or mountain ranges, which have influenced gene flow among populations, as observed in various edible insects in Thailand [9,10]. As mentioned earlier, *S. aequisignata* and *S. ruficornis* prefer different forest types as their habitats. In this case, significant genetic differences were found between *S. aequisignata* populations from northeastern Thailand and other regions, supporting the hypothesis that habitat preferences and ecological barriers have contributed to genetic differentiation. This differentiation may be driven by variations in forest types and ecological conditions.

Previous evidence has shown that genetic differences exist among *A. viridis* lineages associated with distinct host plants, revealing hidden diversity and suggesting that host specialization could be a driving force behind speciation in this jewel beetle [7]. Similarly, the genetic variation and divergence observed in this study may have been influenced by differences in host trees or ecological habitats. However, this hypothesis requires further investigation. Moreover, some populations in this study that were geographically distant from each other exhibited significant genetic differences, suggesting that spatial distance may contribute to genetic divergence. Similar patterns have been observed in other beetles, such as saproxylic beetles [25] and dung beetles [26]. To better understand the relationship between genetic differentiation and landscape isolation, future studies should test hypotheses related to isolation-by-distance [27], isolation-by-barrier [28], and isolation-by-resistance [29]. Additionally, testing phylogenetic signals in habitat selection or responses to environmental factors is necessary to gain insight into the evolutionary history shaping these patterns [30].

Notably, a sample of the *S. aequisignata* haplotype GgC45 from Loei Province did not fit into any haplogroup in the haplotype network analysis or genetic lineage delineation analyzed by the PTP approach, suggesting the presence of another highly divergent lineage, possibly a cryptic species in Loei Province. Further investigations with a larger sample size from Loei and adjacent areas are needed to clarify this genetic distinction.

Human activities, particularly trade, may influence the gene flow of jewel beetles by facilitating their movement across biogeographical barriers. The collection and sale of beetles for ornamental purposes may introduce individuals from different populations into new regions, leading to genetic exchange that would not naturally occur. Genetic differentiation among populations of these beetles may be shaped by trade practices that inadvertently transport insects across natural barriers such as mountains and rivers. For example, the large brown cricket trade along the Thailand–Lao PDR border across the Mekong River [8,31] and giant water bugs along the Thai–Myanmar border [9,32] suggests that gene flow may occur via human-mediated movement, facilitating gene flow.

The high genetic variation and presence of unique haplotypes in *S. aequisignata* and *S. ruficornis* in this study provide valuable basic information for the genetic conservation and potential development of jewel beetle farming. The significant genetic differences detected in natural populations could be useful for selecting suitable breeding pairs in captive breeding programs, thereby maximizing genetic diversity in farmed populations [33]. The observed genetic differences between the populations of these jewel beetles are extremely important for their successful conservation in the future. For instance, understanding the genetic structure of different populations can help identify distinct genetic lineages that require targeted conservation strategies. Conservation efforts should focus on preserving these unique genetic variations by maintaining habitat connectivity and preventing population fragmentation. Additionally, conservation programs should prioritize the protection of habitats that harbor populations with unique haplotypes to ensure the long-term survival of these species in the wild [34].

However, the *CO1* and 16S rDNA genes used as genetic markers in this study may not be the most suitable for detecting genetic differentiation and genetic structure in *S. ruficornis*. In contrast, *CO1* sequences appear to be effective for investigating intraspecific genetic variation in *S. aequisignata* due to their high nucleotide variability. The development of additional highly polymorphic genetic markers, such as non-coding regions (intron) or microsatellite DNA, could provide a clearer understanding of the genetic variation, population structure, and evolutionary history of these jewel beetles in Thailand, Lao PDR, and other endemic regions of Southeast Asia.

## 5. Conclusions

This study provides the first comprehensive genetic assessment of *S. aequisignata* and *S. ruficornis* in Thailand and Lao PDR, revealing high genetic diversity and significant genetic structuring among populations. Our findings suggest that while these two species exhibit distinct habitat preferences, their distributions partially overlap, indicating that their ecological separation is not absolute. The detection of three haplogroups within *S. aequisignata* suggests how historical biogeographical barriers, such as mountains and rivers, along with human-mediated trade, may have influenced gene flow and genetic differentiation, highlighting the need for conservation strategies. Future research incorporating additional genetic markers and broader sampling sites will enhance our understanding of these species’ evolutionary history and support sustainable management efforts.

## Figures and Tables

**Table 1:** Details of sampling localities of *Sternocera ruficornis* and *S. aequisignata* from Thailand and Lao PDR, with the number of examined samples analyzed using 16S rDNA and *CO1* gene.

Country	Province	District	Code	Region	16S rDNA		*CO1*	
					RG ^a^	GG ^b^	RG	GG
THAILAND	Nakhon Sawan	Banphot Phisai	NSN	Central	-	2	-	2
	Lopburi	Chaibadan	LRI	Central	-	12	-	10
	Kampang Phet	Khanu Woralaksaburi	KPT	North	-	10	-	7
		Mueang	KPM	North	3	-	3	-
	Phetchabun	Lom Sak	PBI	North	-	14	-	7
	Kanchanaburi	Si Sawat	KRI	West	-	12	-	10
	Chaiyaphum	Mueang	CPM	Northeast	7	-	4	-
	Kalasin	Khao Wong	KSK	Northeast	7	-	7	1
		Huai Mek	KSM	Northeast	8	-	6	-
		Huai Phueng	KSP	Northeast	9	-	9	-
		Baukhao	KSB	Northeast	2	-	-	-
	Khon Kaen	Khao Suan Kwang	KKN	Northeast	5	-	2	-
	Loei	Phu Ruea	LEI	Northeast	-	1	-	1
	Mukdahan	Kamcha-i	MDH	Northeast	3	-	3	-
		Dontan	MDD	Northeast	5	-	5	-
	Nakhon Phanom	Mueang	NPM	Northeast	11	-	8	-
	Nong Bua Lam Phu	Non Sang	NBP	Northeast	9	-	3	-
	Nong Khai	Tha Bo	NKI	Northeast	11	-	6	-
	Roi Et	Pho Chai	RET	Northeast	9	-	4	-
		Suwannaphumm	RES	Northeast	1	2	-	2
	Sakon Nakhon	Phu Phan	SKN	Northeast	7	-	4	-
	Ubon Ratchathani	Khemarat	UBK	Northeast	8	-	6	-
		Khong Chiam	UBC	Northeast	8	13	9	14
		Nam Khun	UBN	Northeast	9	-	7	-
	Udonthani	Mueang	UDN	Northeast	19	3	13	3
		Wang Sam Mo	UDW	Northeast	-	-	10	-
LAO PDR	Khammouan	Tha Khek	KMT	South	9	4	7	3
	Svannakhet	Songkon	SVS	South	11	7	9	7
Total					161	80	125	67

^a^ red-legged *S. ruficornis*; ^b^ green-legged *S. aequisignata.*

**Table 2:** Molecular diversity indices of *Sternocera aequisignata* from different geographical localities in Thailand and Lao PDR based on 16S rDNA and *CO1* sequences analyses.

Populations	*CO1*						16S rDNA					
	*n*	S	H	Uh	Hd ± SD	Nd ± SD	*n*	S	H	Uh	Hd ± SD	Nd ± SD
NSN	2	2	2	2	1.000 ± 0.500	0.0032 ± 0.0016	2	1	2	0	1.000 ± 0.500	0.0021 ± 0.0011
LRI	10	13	7	3	0.911 ± 0.077	0.0066 ± 0.0013	12	13	8	3	0.924 ± 0.057	0.0063 ± 0.0019
KPT	7	26	7	7	1.000 ± 0.076	0.0143 ± 0.0014	10	11	7	5	0.911 ± 0.077	0.0070 ± 0.0010
PBI	7	7	4	4	0.810 ± 0.130	0.0053 ± 0.0009	14	5	3	3	0.484 ± 0.142	0.0028 ± 0.0010
KRI	10	39	8	4	0.956 ± 0.059	0.0236 ± 0.0047	12	13	7	2	0.894 ± 0.063	0.0087 ± 0.0015
RES	2	9	2	2	1.000 ± 0.500	0.0144 ± 0.0072	2	1	2	1	1.000 ± 0.500	0.0021 ± 0.0011
UBC	14	29	8	7	0.890 ± 0.060	0.0134 ± 0.0013	13	5	7	5	0.885 ± 0.064	0.0032 ± 0.0005
UDN	3	7	3	3	1.000 ± 0.272	0.0074 ± 0.0027	3	3	3	2	1.000 ± 0.272	0.0042 ± 0.0015
KMT	3	7	3	3	1.000 ± 0.272	0.0074 ± 0.0023	4	2	3	3	0.833 ± 0.222	0.0025 ± 0.0008
SVS	7	51	4	4	0.714 ± 0.181	0.0324 ± 0.0092	7	9	4	1	0.714 ± 0.181	0.0088 ± 0.0020
Total	67	105	45	39	0.983 ± 0.006	0.0361 ± 0.0020	80	29	35	25	0.958 ± 0.010	0.0116 ± 0.0004

*n*, sample size; S, segregation site; H, number of haplotypes; Uh, unique haplotype; Hd, haplotype diversity; Nd, nucleotide. diversity; SD, standard deviation. *S. aequisignata* from LEI and KSK were not included in this table because only one sample was available. Locality codes are provided in Table 1.

**Table 3:** Molecular diversity indices of *Sternocera ruficornis* from different geographical localities in Thailand and Lao PDR based on 16S rDNA and *CO1* sequences analyses.

Populations	*CO1*						16S rDNA					
	*n*	S	H	Uh	Hd ± SD	Nd ± SD	*n*	S	H	Uh	Hd ± SD	Nd ± SD
KPM	3	4	2	2	0.667 ± 0.314	0.0043 ± 0.0020	3	0	1	0	0	0
CPM	4	13	2	2	0.667 ± 0.204	0.0138 ± 0.0042	7	0	1	0	0	0
KSK	7	8	6	5	0.952 ± 0.096	0.0040 ± 0.0009	7	2	2	1	0.286 ± 0.196	0.0012 ± 0.0008
KSM	6	16	2	1	0.333 ± 0.215	0.0085 ± 0.0055	8	2	3	2	0.607 ± 0.164	0.0014 ± 0.0005
KSP	9	13	9	7	1.000 ± 0.052	0.0056 ± 0.0010	10	0	1	0	0	0
KSB	2	11	2	0	1.000 ± 0.500	0.0175 ± 0.0088	2	2	2	1	1.000 ± 0.500	0.0042 ± 0.0021
KKN	2	3	2	2	1.000 ± 0.500	0.0048 ± 0.0024	5	0	1	1	0	0
MDH	3	3	3	2	1.000 ± 0.272	0.0032 ± 0.0009	3	0	1	0	0	0
MDD	5	15	3	1	0.800 ± 0.164	0.0099 ± 0.0053	6	4	3	1	0.600 ± 0.215	0.0028 ± 0.0014
NPM	8	1	2	1	0.250 ± 0.180	0.0004 ± 0.0003	11	0	1	0	0	0
NBP	3	3	3	3	1.000 ± 0.272	0.0032 ± 0.0011	9	2	3	3	0.556 ± 0.165	0.0013 ± 0.0005
NKI	6	10	4	2	0.800 ± 0.172	0.0067 ± 0.0022	11	1	2	0	0.436 ± 0.133	0.0009 ± 0.0003
RET	4	23	3	2	0.833 ± 0.222	0.0186 ± 0.0089	9	5	3	1	0.417 ± 0.191	0.0027 ± 0.0014
SKN	4	3	2	1	0.667 ± 0.204	0.0032 ± 0.0010	7	2	3	1	0.524 ± 0.209	0.0012 ± 0.0005
UBK	6	20	4	1	0.867 ± 0.129	0.0168 ± 0.0050	8	1	2	1	0.250 ± 0.180	0.0005 ± 0.0004
UBC	9	26	8	7	0.972 ± 0.064	0.0120 ± 0.0038	11	8	6	5	0.727 ± 0.144	0.0034 ± 0.0010
UBN	7	13	7	6	1.000 ± 0.076	0.0087 ± 0.0021	9	4	5	4	0.861 ± 0.087	0.0028 ± 0.0006
UDN	13	13	8	6	0.923 ± 0.050	0.0064 ± 0.0006	19	4	5	3	0.591 ± 0.118	0.0015 ± 0.0004
UDW	10	5	3	1	0.378 ± 0.181	0.0016 ± 0.0008	n/a	n/a	n/a	n/a	n/a	n/a
KMT	7	1	2	1	0.286 ± 0.196	0.0005 ± 0.0003	9	0	1	0	0	0
SVS	9	1	2	1	0.222 ± 0.166	0.0004 ± 0.0003	11	1	2	1	0.182 ± 0.144	0.0004 ± 0.0003
Total	127	81	62	54	0.947 ± 0.012	0.0174 ± 0.0007	166	29	28	25	0.706 ± 0.029	0.0030 ± 0.0003

*n*, sample size; S, segregation site; H, number of haplotypes; Uh, unique haplotype; Hd, haplotype diversity; Nd, nucleotide diversity; SD, standard deviation; n/a, not applicable. Locality codes are provided in Table 1.

**Table 4:** Analysis of Molecular Variance (AMOVA) based on *CO1* sequences of the green-legged *Sternocera aequisignata* populations defined by three genetic groups (GG1–GG3).

Source of Variation	d.f.	Sum of Squares	Variance Components	Percentage of Variation	Fixation Index
Among groups	2	479.653	12.1708	73.06	*F*_CT_ = 0.73059 *
Among populations within groups	10	88.739	1.3709	8.23	*F*_SC_ = 0.30545 *
Within populations	53	165.214	3.11725	18.71	*F*_ST_ = 0.81288 *

d.f., degree of freedom; * *p*-value < 0.001.

## Data Availability

All data are available upon request.

## Supplementary Materials

The following supporting information can be downloaded at https://www.mdpi.com/article/10.3390/insects16030322/s1, Table S1: Variable nucleotide positions of *CO1* sequences; Table S2: Variable nucleotide positions of 16S rDNA sequences; Table S3: Neutrality test of *Sternocera aequisignata* (Gg) and *S. ruficornis* (Rg) populations based on CO1 and 16S rDNA sequences; Table S4: Genetic differences (*p*-distance) calculated by *CO1* sequences; Table S5: Genetic differences (*p*-distance) calculated by 16S rDNA sequences; Figure S1: Minimum spanning haplotype network constructed from 16S rDNA haplotypes; Figure S2: Phylogenetic tree constructed from 16S rDNA sequences.
