# Supplementary material for: Exploring the Genetic Diversity of the Jewel Beetles Sternocera aequisignata Saunders, 1866, and S. ruficornis Saunders, 1866 (Coleoptera: Buprestidae) in Thailand and Lao PDR
Source: Insects. 2025 Mar 19;16(3):322. doi: 10.3390/insects16030322 (PMC11942929; doi:10.3390/insects16030322)
Supplement: Supplementary file 1 [file insects-16-00322-s001.zip › Table S4.pdf]

Table S4. Genetic differences *p*-distance calculated based on *CO1* (lower triangle) and 16S rDNA (upper triangle) compare among different populations of *Sternocera aequisignata*.

Code	RES	KPT	NSN	UBC	UDN	SVS	KMT	LRI	PBI	KRI	LEI
**RES**	-	0.0088	0.0084	0.0151	0.0025	0.0069	0.0037	0.0082	0.0083	0.0081	0.0116
**KPT**	0.0175	-	0.0057	0.0176	0.0088	0.0127	0.0111	0.0073	0.0150	0.0083	0.0154
**NSN**	0.0144	0.0107	-	0.0168	0.0088	0.0125	0.0089	0.0044	0.0140	0.0065	0.0158
**UBC**	0.0579	0.0591	0.0575	-	0.0134	0.0102	0.0142	0.0181	0.0167	0.0181	0.0206
**UDN**	0.0104	0.0128	0.0096	0.0551	-	0.0068	0.0051	0.0085	0.0089	0.0082	0.0112
**SVS**	0.0275	0.0303	0.0280	0.0435	0.0238	-	0.0077	0.0129	0.0108	0.0126	0.0147
**KMT**	0.0215	0.0278	0.0245	0.0627	0.0214	0.0326	-	0.0105	0.0067	0.0107	0.0142
**LRI**	0.0155	0.0113	0.0072	0.0585	0.0107	0.0281	0.0248	-	0.0147	0.0074	0.0153
**PBI**	0.0440	0.0485	0.0447	0.0520	0.0429	0.0474	0.0514	0.0476	-	0.0140	0.0114
**KRI**	0.0219	0.0207	0.0172	0.0571	0.0182	0.0326	0.0302	0.0171	0.0378	-	0.0154
**LEI**	0.0287	0.0349	0.0319	0.0570	0.0266	0.0371	0.0372	0.0340	0.0392	0.0341	-
**KSK**	0.0526	0.0560	0.0542	0.0105	0.0516	0.0410	0.0590	0.0544	0.0488	0.0528	0.0526

Population codes are provided in Table 1.
