# Supplementary material for: Exploring the Genetic Diversity of the Jewel Beetles Sternocera aequisignata Saunders, 1866, and S. ruficornis Saunders, 1866 (Coleoptera: Buprestidae) in Thailand and Lao PDR
Source: Insects. 2025 Mar 19;16(3):322. doi: 10.3390/insects16030322 (PMC11942929; doi:10.3390/insects16030322)
Supplement: Supplementary file 1 [file insects-16-00322-s001.zip › Table S5.pdf]

Table S5. Genetic differences *p*-distance calculated based on *CO1* (lower triangle) and 16S rDNA (upper triangle) compared among different populations of *Sternocera ruficornis*.

Code	SKN	CPM	RET	KSK	KSP	KSM	UBK	UBC	UBN	KKN	KPM	KSB	MDH	MDD	UDN	SVS	KMT	NKI	NPM	NBP	RES
**SKN**	-	0.0021	0.0035	0.0012	0.0006	0.0029	0.0024	0.0038	0.0108	0.0042	0.0021	0.0024	0.0006	0.0029	0.0013	0.0023	0.0021	0.0010	0.0021	0.0049	0.0021
**CPM**	0.0159	-	0.0014	0.0027	0.0021	0.0008	0.0003	0.0017	0.0087	0.0021	0.0000	0.0021	0.0021	0.0014	0.0022	0.0002	0.0000	0.0015	0.0000	0.0028	0.0000
**RET**	0.0211	0.0171	-	0.0041	0.0035	0.0022	0.0016	0.0030	0.0080	0.0035	0.0014	0.0035	0.0035	0.0028	0.0036	0.0016	0.0014	0.0029	0.0014	0.0042	0.0014
**KSK**	0.0040	0.0155	0.0210	-	0.0006	0.0035	0.0030	0.0044	0.0114	0.0048	0.0027	0.0027	0.0006	0.0034	0.0014	0.0029	0.0027	0.0012	0.0027	0.0055	0.0027
**KSP**	0.0052	0.0158	0.0216	0.0046	-	0.0029	0.0024	0.0038	0.0108	0.0042	0.0021	0.0021	0.0000	0.0028	0.0008	0.0023	0.0021	0.0006	0.0021	0.0049	0.0021
**KSM**	0.0191	0.0144	0.0125	0.0190	0.0197	-	0.0011	0.0025	0.0094	0.0029	0.0008	0.0024	0.0029	0.0022	0.0030	0.0010	0.0008	0.0023	0.0008	0.0036	0.0008
**UBK**	0.0221	0.0247	0.0250	0.0223	0.0228	0.0204	-	0.0020	0.0084	0.0024	0.0003	0.0024	0.0024	0.0017	0.0025	0.0005	0.0003	0.0018	0.0003	0.0031	0.0003
**UBC**	0.0139	0.0237	0.0253	0.0146	0.0155	0.0220	0.0209	-	0.0101	0.0038	0.0017	0.0038	0.0038	0.0031	0.0039	0.0019	0.0017	0.0033	0.0017	0.0045	0.0017
**UBN**	0.0224	0.0265	0.0255	0.0221	0.0227	0.0303	0.0339	0.0250	-	0.0108	0.0087	0.0108	0.0108	0.0101	0.0109	0.0088	0.0087	0.0102	0.0087	0.0115	0.0087
**KKN**	0.0128	0.0175	0.0227	0.0124	0.0123	0.0221	0.0268	0.0219	0.0229	-	0.0021	0.0042	0.0042	0.0035	0.0043	0.0023	0.0021	0.0036	0.0021	0.0047	0.0021
**KPM**	0.0136	0.0181	0.0233	0.0134	0.0139	0.0216	0.0248	0.0213	0.0258	0.0157	-	0.0021	0.0021	0.0014	0.0022	0.0002	0.0000	0.0015	0.0000	0.0028	0.0000
**KSB**	0.0112	0.0128	0.0144	0.0108	0.0116	0.0106	0.0205	0.0183	0.0254	0.0159	0.0165	-	0.0021	0.0032	0.0025	0.0023	0.0021	0.0021	0.0021	0.0049	0.0021
**MDH**	0.0040	0.0152	0.0206	0.0036	0.0044	0.0186	0.0223	0.0144	0.0213	0.0109	0.0133	0.0104	-	0.0028	0.0008	0.0023	0.0021	0.0006	0.0021	0.0049	0.0021
**MDD**	0.0187	0.0271	0.0297	0.0190	0.0194	0.0258	0.0130	0.0177	0.0321	0.0254	0.0237	0.0225	0.0191	-	0.0030	0.0016	0.0014	0.0024	0.0014	0.0042	0.0014
**UDN**	0.0063	0.0166	0.0216	0.0059	0.0068	0.0194	0.0231	0.0158	0.0237	0.0142	0.0138	0.0121	0.0059	0.0203	-	0.0024	0.0022	0.0012	0.0022	0.0050	0.0022
**SVS**	0.0217	0.0297	0.0313	0.0223	0.0227	0.0270	0.0108	0.0179	0.0341	0.0281	0.0268	0.0249	0.0225	0.0053	0.0232	-	0.0002	0.0017	0.0002	0.0030	0.0002
**KMT**	0.0218	0.0297	0.0313	0.0223	0.0227	0.0271	0.0109	0.0179	0.0342	0.0281	0.0268	0.0249	0.0226	0.0053	0.0233	0.0004	-	0.0015	0.0000	0.0028	0.0000
**NKI**	0.0077	0.0175	0.0225	0.0071	0.0080	0.0200	0.0233	0.0162	0.0252	0.0149	0.0143	0.0130	0.0072	0.0206	0.0067	0.0233	0.0234	-	0.0015	0.0043	0.0015
**NPM**	0.0217	0.0297	0.0313	0.0223	0.0227	0.0270	0.0108	0.0179	0.0341	0.0281	0.0268	0.0249	0.0225	0.0053	0.0233	0.0004	0.0004	0.0233	-	0.0028	0.0000
**NBP**	0.0077	0.0136	0.0191	0.0074	0.0080	0.0179	0.0222	0.0171	0.0205	0.0072	0.0117	0.0114	0.0069	0.0203	0.0090	0.0230	0.0231	0.0095	0.0231	-	0.0028
**UDW**	0.0070	0.0167	0.0216	0.0063	0.0070	0.0187	0.0225	0.0151	0.0247	0.0150	0.0130	0.0120	0.0062	0.0199	0.0049	0.0227	0.0227	0.0045	0.0227	0.0099	-

Population codes are provided in Table 1.
