# Supplementary figures and images for: Exploring the Genetic Diversity of the Jewel Beetles Sternocera aequisignata Saunders, 1866, and S. ruficornis Saunders, 1866 (Coleoptera: Buprestidae) in Thailand and Lao PDR
Source: Insects. 2025 Mar 19;16(3):322. doi: 10.3390/insects16030322 (PMC11942929; doi:10.3390/insects16030322)

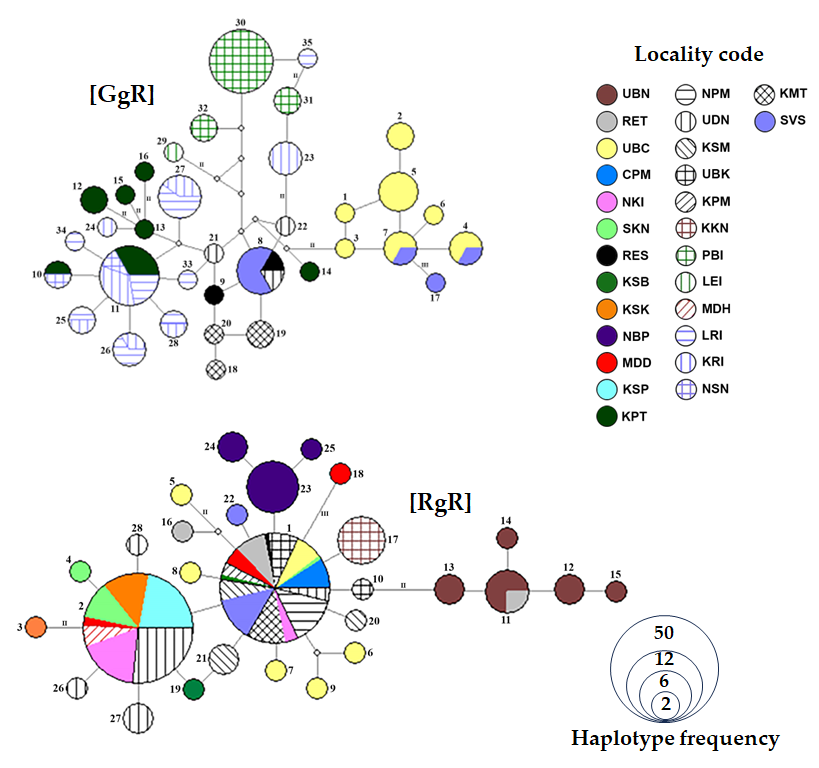

Supplement: Supplementary file 1 [file insects-16-00322-s001.zip › Figure S1.tif]
